# The Way Ahead: Lessons Learned from Decades of Cancer Research on Thymomas and Thymic Carcinomas

**DOI:** 10.3390/cancers16051040

**Published:** 2024-03-04

**Authors:** Philipp Ströbel, Alexander Marx

**Affiliations:** Institute of Pathology, University Medical Center Göttingen, D-37075 Göttingen, Germany; alexander.marx@med.uni-goettingen.de

The history of thymoma (TH) research begins in the early 20th century, when Bell first recognized the epithelial nature of these tumors and their association with myasthenia gravis (MG) [1]. Generations of scientists have been fascinated by the fact that these tumors do not only pose an oncological challenge by infiltrating important thoracic structures or by metastasizing to extrathoracic sites but can also profoundly perturb the immune system and induce autoimmunity. Much later, in 1978, Levine and Rosai were the first to separate thymic carcinomas (TCs) from TH [2]. Since then, the work of many eminent pathologists has helped to define the recognized TH and TC entities (thymic epithelial tumors, TETs) now listed in the current World Health Organization (WHO) classification system [3]. TETs share the fate of many “orphan” diseases with little attention from the public and the pharmaceutic industry with repercussions on the ability to recruit qualified researchers, to publish in high-impact journals, to obtain public funding, and to perform clinical trials. Successful cancer research in other fields has typically followed a structured translational chain from basic research to preclinical translation followed by clinical translation and clinical trials (Figure 1).

TET research in the past has too often suffered from the fact that it has addressed only parts of the translational chain without much impact on clinical practice. There has been comparatively little basic research on TETs and our knowledge on the composition and biology of these rare tumors is still disturbingly incomplete with many open questions; for example, the causes of TETs and predisposing environmental or genetic risk factors are completely unknown. A better understanding of how thymomas elicit autoimmunity could at the same time also help to understand how the thymus shapes the self-tolerant immune repertoire under normal conditions. Although the characteristic recurrent chromosomal alterations have been known for decades, their functional consequences are not fully understood and merit further study. The relative frequency of these alterations correlates well with clinical aggressiveness, but the factors driving malignant behavior, especially in TH, are still unknown. One of the biggest obstacles in TET basic research is the current lack of suitable cell or animal models. The establishment of ex vivo or permanent cell cultures has proven very difficult and frustrating and there are only a couple of published cell lines available worldwide (Table 1). There are currently no published protocols on how to improve the success rate.

The identification of the *GTF2I* mutation [11] and the subsequent establishment of mouse models [12,13] can be considered a milestone because it provides the first experimentally proven evidence of a thymus-specific and functionally relevant gene alteration that explains important aspects of thymoma biology (i.e., impaired terminal maturation of thymic epithelial progenitors with implications for T-cell maturation) and recapitulates the human disease in mice, including the fact that TH develop in aged but not in young mice. The identification of similar mechanisms driving the pathogenesis of the more aggressive TET subtypes and the establishment of suitable in vitro or in vivo model systems for the functional study of these tumors would be similarly groundbreaking and deserve the highest priority.

One of the best options to explore the driving mechanisms behind TETs is the database of the Cancer Genome Atlas Program (TCGA). The TCGA study has been another milestone in the history of TET research because it created an authoritative source of high-quality molecular data and has underpinned the basic concept of the WHO classification [14]. However, the wealth of information contained in the TCGA database has not yet been fully exploited. These data, either at the level of defined single pathways or at the level of multi-omics, can help to reveal unknown molecular mechanisms and could even be used for in silico drug screenings [15]. As an example, the recent identification of a tuft cell-like signature in thymic squamous cell carcinomas [16] has opened a completely new view on TC with potential therapeutic consequences. The tuft-like phenotype in TC is also a good example because the same signature has been identified in other, much more common, cancers in the TCGA collection that are easier to study experimentally. Analogous results obtained in these tumors could eventually be translated to TETs. Findings based on data analyses require validation by functional assays in vitro and in vivo. This caveat may sound trivial, but the recent TET literature shows several examples of publications using highly sophisticated bioinformatic methods leading to potentially relevant observations that, however, will probably be lost without systematic follow-up studies along the translational chain (Figure 1). We believe that the best way to achieve meaningful results is through hypothesis-driven research from interdisciplinary and interprofessional teams that include data scientists and molecular biologists, as well as clinicians (pathologists, radiologists, oncologists, surgeons, etc.) experienced in diagnosing and treating TETs.

As discussed above, preclinical research has been severely hampered by both the lack of representative cell or animal models and by the incomplete picture of the driving mechanisms behind TET biology. However, there are several experimental approaches that can be used to circumvent this problem and still address relevant questions. In one study, phosphoproteomic data from TET tissues were used to first define a sunitinib-response prediction algorithm, which was then applied to cell lines from other tumors (e.g., colorectal or lung) for functional validation. Freshly isolated TH tumor cells, short-term ex vivo cell TH cultures [7,17], and living tissue slices [18] have been successfully used, e.g., for BH3 profiling to interrogate the mitochondrial apoptosis pathway in cells [19] or unbiased drug screening assays [8,20,21].

Ideally, these preclinical studies should help provide sufficient evidence to initiate clinical trials. Based on the published literature, one is tempted to hypothesize that many of the few clinical trials in TETs, even the successful ones, were mostly based on empirical data or analogies to other tumors rather than on robust preclinical evidence. The inherent danger of this approach lies in the possibility that some trials may have failed only due to confounding factors such as tumor heterogeneity, lack of predictive biomarkers, and thus, suboptimal patient selection.

Arguably one of the most important milestones in TET research of the last 30 years has been the foundation of the International Thymic Malignancy Interest Group (ITMIG) in 2010. For the first time in history, ITMIG has brought together TET patients and their families and international experts from all disciplines involved in TET research, diagnosis, and treatment. Visible outputs of this effort were multiple highly successful conferences, the largest TET database worldwide [22], and a number of important consensus papers and proposals [23,24], which, among other achievements, have helped to lay the basis for the first TNM classification system of TETs [25]. ITMIG and other prominent consortia, such as the French RYTHMIC network [26], have demonstrated the impact that national or international efforts with own funding can achieve. These networks have greatly helped to promote public awareness, create solid data and case collections, and, in the case of RYTHMIC, have even initiated their own clinical trials [27]. The biggest advantage of such networks is their ability to coordinate focused interdisciplinary programs along the translational chain that have the power to prioritize and answer clinically relevant questions in a systematic fashion.

For this Special Issue of *Cancers*, we asked experts in the field for their views on the current state of TET research and treatment, and their recommendations for what could be improved and where the field should go in the future. With the availability of high-quality data sets, comprehensive bioinformatic analyses, and cutting-edge techniques such as single-cell analysis [28] and high-throughput siRNA or drug screening, the opportunities to improve our knowledge of TETs and translate it into more effective treatments have never been better. It is in the hands of the scientific community to orchestrate our efforts for the benefit of our patients.

## Figures and Tables

**Figure 1:**
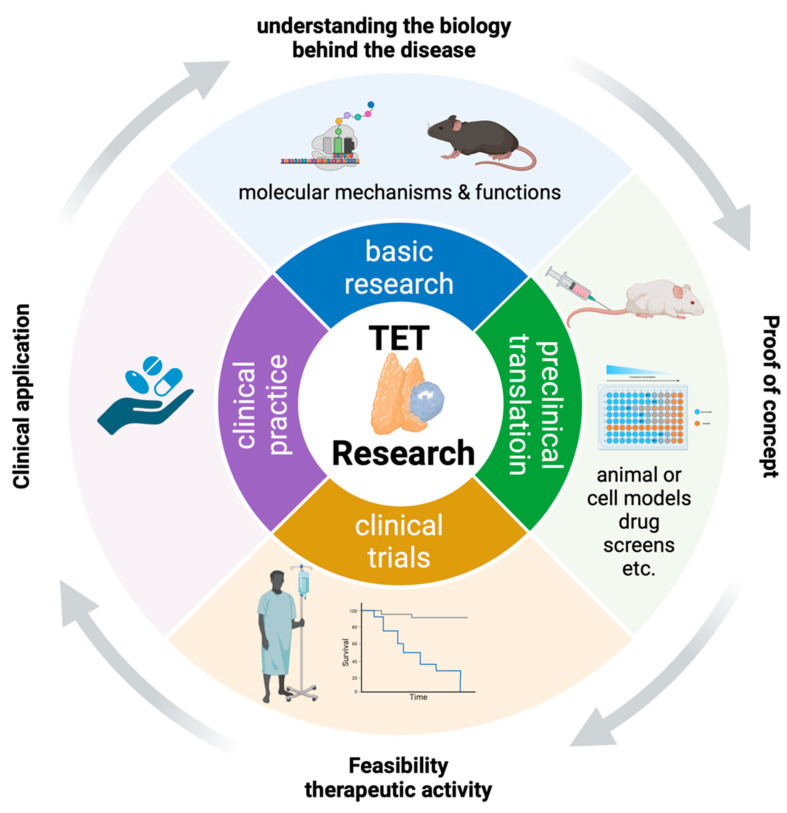
Translational chain from basic research to clinical application.

**Table 1:** List of publicly available thymoma and thymic carcinoma cell lines.

Cell Line	Initially Derived from	Accession No *	Reference
IU-TAB-1	Type AB thymoma	RRID:CVCL_D551	[4]
T68	Type AB thymoma	RRID:CVCL_C6JV	[5]
Thy0517	Type AB thymoma with MG	RRID:CVCL_2Z85	[6]
Thy0517	Type AB thymoma with MG	RRID:CVCL_2Z85	[7]
T1682	Type B1 thymoma	RRID:CVCL_D023	[8]
T1889	Thymic carcinoma	RRID:CVCL_D024	[8]
MP57	Thymic carcinoma	RRID:CVCL_VJ89	[9]
ThyL-6	Thymic carcinoma	RRID:CVCL_2Z86	[10]
Hs 702.T	Thymic neoplasm (unspecified)	(RRID:CVCL_S306)	

* cellosaurus.org; MG: myasthenia gravis.
